# Humanoid Robots perform cognitive assessments: to address healthcare worker shortages

**DOI:** 10.1002/alz.088116

**Published:** 2025-01-09

**Authors:** Arshia A Khan

**Affiliations:** ^1^ University of Minnesota Duluth, Duluth, MN USA

## Abstract

**Background:**

The rising demand for alternative dementia assessments, fueled by healthcare workforce shortages and the growing population of individuals affected with dementia, necessitates innovative approaches to address accessibility, logistics, and diverse populations. The utilization of robots in cognitive assessments emerges as a promising solution, promising efficiency and engagement, while navigating the complex landscape of dementia care challenges.

**Method:**

Existing cognitive assessment tools were examined to develop a humanoid robot to deliver cognitive assessment. This robot‐assisted cognitive assessment development involved integrating artificial intelligence with robotic technology to create a comprehensive tool. Initially, the design phase included defining the cognitive domains to be assessed and tailoring the assessment tasks to suit robot‐human interaction. The robot’s programming incorporated adaptive features to respond to individual variations in cognitive abilities. Sensory modules, such as cameras and microphones, in the robot were integrated for real‐time data collection, enabling the robot to observe and interact with the individual. Iterative testing and refinement were performed to ensure the accuracy and reliability of the robot‐assisted cognitive assessment tool. Throughout the development process emphasis was on creating a robust and user‐friendly system that aligns with the nuanced nature of cognitive assessments for individuals affected by dementia.

**Result:**

Robot‐assisted cognitive assessments were tested in a nursing home setting, showing promising results and providing valuable insights into residents' cognitive functioning.

**Conclusion:**

The robot assisted assessments, integrated with AI, offer a comprehensive understanding of memory, attention, and other cognitive domains. The individual variability in cognitive abilities among residents is highlighted, enabling personalized care plans tailored to specific needs. The positive engagement and usability reported by residents suggest a potential avenue for integrating this technology into routine care practices. The real‐time data collection capabilities of the robot contribute to a more dynamic and accurate assessment of cognitive health. Caregivers benefit from the objective cognitive performance data, allowing for timely interventions and adjustments in care plans. The iterative feedback loop from residents and caregivers facilitates continuous improvement in the robot‐assisted cognitive assessment tool, making it a valuable and beneficial asset in enhancing the quality of care provided in nursing homes.

